# Biological Studies on Alcohol-Induced Neuronal Damage

**DOI:** 10.4306/pi.2008.5.1.21

**Published:** 2008-03-31

**Authors:** Masaru Tateno, Toshikazu Saito

**Affiliations:** Department of Neuropsychiatry, Sapporo Medical University, Sapporo, Japan.

**Keywords:** Alcohol, Alcoholism, Fetal alcohol syndrome, Neural stem cell, Neurogenesis

## Abstract

Alcohol is a well-known cytotoxic agent which causes various kinds of neuronal damage. In spite of thousands of published studies, the true mechanism of alcohol-induced neuronal damage remains unclear. Neurogenesis is the generation of neurons from neural stem cells (NSCs) and occurs in predominantly two regions of the brain, the subventricular zone and the dentate gyrus of the hippocampus. NSCs are the self-renewing, multipotent precursor cells of neurons, astrocytes, and oligodendrocytes in the central nervous system. Recent studies have begun to illuminate the role of neurogenesis in the biological and cellular basis of psychiatric disorders and several clinical symptoms seen in alcoholism such as depression, cognitive impairment, underlying stress and brain atrophy have been linked to impaired neurogenesis. Heavy alcohol consumption decreases neurogenesis in animals, while *in vitro* studies have shown decreased generation of new neurons after alcohol exposure. These findings suggest that decreased neurogenesis is important in the pathophysiology of alcoholism. Neurogenesis can be divided into four stages; proliferation, migration, differentiation and survival. Our *in vitro* studies on NSCs showed that alcohol decreased neuronal differentiation at doses lower than those that affected cell survival and suggested that neuron-restrictive silencer factor, or repressor element-1 silencing transcription factor (NRSF/REST) could be involved in alcohol-induced inhibition of neuronal differentiation. In an animal model of fetal alcohol effects behavioral symptoms improved after NSC transplantation. Neurogenesis could be the target for new strategies to treat alcohol related disorders.

## Introduction

Alcohol is a well-known deleterious agent which damages various organs and causes serious health problems. From early times alcohol abuse has been a serious social problem. The World Health Organization estimates that worldwide about 140 million people suffer from alcoholism (http://www.who.int/en/). Despite thousands of studies designed to elucidate the underlying mechanism of alcohol dependence, its pathophysiology remains obscure. Recently, impaired neurogenesis has been considered the most plausible cause of alcohol related disorders and is attracting great attention in the field of alcohol research.

The long-standing dogma that new neurons cannot be produced in adult brains^1^ was overturned by breakthrough studies by Reynolds and Weiss^2^ in which cells from the striatum of adult mice were induced to proliferate *in vitro*. Subsequently, Eriksson et al.^3^ using immunofluorescent labeling for bromodeoxyuridine (BrdU), demonstrated that new neurons were generated from progenitor cells in the dentate gyrus of the adult human brain indicating that cells in the human hippocampus retained the capacity to give rise to neurons throughout life. These concept-breaking findings triggered many more studies of neurogenesis, the phenomenon of new neuron generation in the central nervous system (CNS). Every mammal investigated to date including humans has been found to have neurogenesis in specific regions of the brain. Neural stem cells (NSCs) are the origin of newly generated neurons and their function is critical in neurogenesis. Many researchers are deeply interested in the role of NSC and in relations between changes in their function and the pathophysiology of psychiatric disorders. Emerging evidence suggests that altered NSC function plays an important role in the development of alcohol-induced neuronal damage as observed in alcoholism.^4^-^7^

This review includes a brief overview of previous studies on intracellular signaling cascades which relate to alcohol-induced cytotoxicity and summarizes accumulating evidence from studies of neurogenesis which provide new insights into the underlying mechanism of alcohol-related neuro-psychiatric disorders.

## Alcohol and the Intracellular Signaling Cascade

Alcohol consumption activates the reward pathway of the brain and causes euphoria that contributes to the development of alcohol dependency. Chronic and excessive alcohol consumption damages neurons through their intracellular signal transduction pathways. Our postmortem studies using brain samples of cerebral cortex and platelets from alcoholics revealed a significant quantitative decrease of adenylyl cyclase (AC)-I and VIII.^8^ Activation of the G-protein-AC system generates cyclic adenosine monophosphate (AMP) (cAMP) which activates cAMP-dependent protein kinase A (A kinase). A kinase transmigrates into the nucleus, and then phosphorylates a transcription factor named cAMP-responsive element binding protein (CREB). CREB has been reported to have various target genes and to promote their transcription.^9^-^11^ The targets of CREB include neurotrophic factors such as brain-derived neurotrophic factor (BDNF), which are essential for synthesizing neurotransmitters and the molecules necessary for neuron survival. In rats, long-term alcohol consumption decreased CREB activity in the brain striatum.^12^ *In vitro* studies using cultured rat cortical neurons revealed alcohol-induced reduction of CREB activity and decreased expression of BDNF.^13^ These findings suggest that alcohol is a negative regulator of cAMP-CREB signal transduction acting intracellularly. Furthermore, a study using cultured neurons indicated that short-term alcohol treatment (0.5-2 hours) activated the cAMP system and increased expression of BDNF and other factors, whereas more prolonged treatment (>24 hour) inhibited cAMP and decreased BDNF expression.^14^ BDNF and CREB are reported to play an essential role in the pathophysiology of depression^15^ which is the most common comorbidity of alcoholism. Our postmortem study showed a reduced expression of phosphorylated CREB in the orbitofrontal cortex of patients with depression.^16^ This correlation between an altered cAMP-CREB-BDNF cascade and depressive symptoms suggests that in the brains of alcoholics, dysfunction and loss of neurons may be the outcome of altered intracellular signals due to the suppressed CREB activity and the decreased BDNF level.

## Neurogenesis and Psychiatric Disorders

The NSC is a primitive, immature cell able to self-renew and give rise to multilineage progeny of CNS cells such as neurons, astrocytes oligodendrocytes etc.^17^-^19^ NSCs reside in two discrete regions in the brain, the subventricular zone and the dentate gyrus of the hippocampus, and continue to generate new neurons throughout life. NSCs, with their multiple possibilities, have attracted considerable attention from researchers in various scientific fields. With the acceptance of neurogenesis, more studies have focused on its relation to the pathophysiology of neuro-psychiatric disorders includeing alcoholism.

The frequent co-existence of several clinical symptoms such as depression, cognitive impairment and brain atrophy in patients with alcoholism supports the hypothesis that decreased neurogenesis is related to the underlying mechanism of alcoholism. Depression is common in alcoholics^20^ and psychological stressors significantly contribute to the development of alcohol-related problems.^21^,^22^ On the other hand, both physical and psychological chronic stresses are the most common animal model of depression. Stressful events elevate serum glucocorticoid levels and stimulate glutamate release in the hippocampus which inhibits the proliferation of new cells.^23^,^24^

MRI studies demonstrated a reduced hippocampus volume in patients with a long history of recurrent depressive episodes.^25^-^27^ Neuroimaging studies of alcoholics demonstrated decreased brain volumes, especially involving the hippocampus,^28^-^31^ and postmortem findings on alcoholics are consistent with the results from antemortem neuroimaging studies.^32^ The hippocampal volume deficit observed in animal models of alcoholism has been reported to be related to a decrease of neurons in the hippocampus.^33^,^34^ Furthermore, several MRI studies have demonstrated that stress is related to the decreased hippocampal volumes in humans.^35^,^36^ The animal studies describing a decrease of neurons in the hippocampus after chronic stress could, at least partially, explain the lower hippocampal volume seen in patients with PTSD.^37^ In subjects with alcoholism, depression and severe stress, a similar decrease of hippocampal volume was observed.

In regard to the treatment of depression, animal studies have demonstrated that antidepressants,^38^-^40^ mood stabilizers,^41^-^43^ BDNF,^44^,^45^ estrogen,^46^,^47^ electro convulsive treatment (ECT),^48^-^50^ physical exercise^51^-^53^ and enriched environment,^54^,^55^ all increase neurogenesis. *In vivo* studies by Santarelli et al.^56^ suggested that hippocampal neurogenesis is required to exert antidepressive effects as revealed by behavioral analysis. These findings emphasize the important role of neurogenesis in the pathophysiology of depression in several different ways.^57^-^59^

Another common clinical symptom in alcoholism is cognitive dysfunction. Severe alcohol-related dementia is known as Korsakoff's syndrome. Clinical studies on alcoholism have confirmed the effects of alcohol on cognitive functions^60^,^61^ and several animal studies have shown cognitive impairment after chronic alcohol consumption.^62^-^64^ Initially, the relationship of neurogenesis to learning attracted the attention of researchers,^52^,^65^ with the result that the connection between learning/memory and neurogenesis is now widely recognized.^66^,^67^ Predictably, dementia sufferers have a smaller brain mass than age matched controls, especially involving the hippocampus. All these findings emphasize the importance of decreased neurogenesis in the pathophysiology of alcoholism.

## Alcohol-Induced Neuronal Damage and Impaired Neural Stem Cell Function

Alcohol causes various kinds of neuronal damage both to the developing and adult brain.^68^-^72^ Previously, alcohol was reported to decrease the number of neurons by increasing apoptotic cell death and reducing cell proliferation through prolongation of the cell cycle.^73^ More recent studies have implicated disrupted neurogenesis as a mechanism that impairs the neural network as described earlier in this review. Considering the four stages of neurogenesis; proliferation, migration, differentiation and survival, the loss of neurons may reflect not only direct damage, which decreases cell survival, but also a reduction in the number of newly generated neurons through suppression of NSC proliferation and differentiation into neuronal subtypes. The overall loss of neurons might seriously compromise the development and maintenance of the neuronal network.

A study in alcohol dependent rats demonstrated selective inhibition of neurogenesis in the hippocampus and a significant increase of hippocampal neurons after weeks of abstinence.^74^ An *in vitro* study using NSCs prepared from rat embryos on gestational day 13.5 in a monolayer culture^75^,^76^ showed that of the four stages of neurogenesis, differentiation was the most susceptible to alcohol effects under the employed experimental conditions.^77^ Furthermore, alcohol inhibited neuronal differentiation of NSCs at lower concentrations than those that impaired cell survival.^78^ These results suggest that alcohol effects on neuronal differentiation might occur in addition to alcoholinduced neuronal loss due to direct neuronal death and prolonged cell cycle which reduces neurons. Under the same experimental conditions the alcohol inhibition of neuronal differentiation was reduced by the treatment with neurotrophic factors such as BDNF and Insulin-like growth factor I (IGF-I).^77^ Further studies have suggested that these inhibitory effects of alcohol on NSC function involve extracellular regulated kinase (ERK) which is a member of the mitogenactivated protein (MAP) kinase family and plays an important role in transmission of extracellular signals such as the neurotrophic factor signaling cascade.^79^,^80^

The CNS consists of a mixture of neuronal and glial cells and it has long been controversial whether alcohol could affect the direction of NSC differentiation, that is, towards neuronal or glial cells. In some studies, the ratio of neurons to glia was unchanged even after alcohol exposure while other studies reported increased astrocytic differentiation after alcohol exposure.^73^,^81^,^82^ Astrocytes increase in number in response to various insults to the CNS.^83^-^86^ This reactive increase of glial cells following CNS injuries has been considered detrimental to CNS repair. However, some studies have suggested that the newly generated astrocytes in situ might release several cytokines that promote regeneration of the impaired neural network. The fact that neuronal death has often been accompanied by the generation of new neurons nearby might reflect the release of cytokines from dying neurons.^81^,^82^ Thus, it is possible that the increase of astrocytes and oligodendrocytes after alcohol exposure is a compensatory mechanism intended to repair an impaired neural network by promoting neurite outgrowth and increasing the number of newly generated neurons.

To clarify how alcohol affects the direction of NSC differentiation, *in vitro* studies focusing on neuron-restrictive silencer factor, or repressor element-1 silencing transcription factor (NRSF/REST) were performed. NRSF/REST is a zinc-finger transcription factor which is composed of an N-terminal repressor domain, a cluster of eight zinc fingers that functions as a DNA-binding domain, a highly basic region, a repeat-region and a C-terminal repressor domain with a single zinc finger motif.^87^,^88^ NRSF/REST binds to its target which is by consensus identified as neuron-restrictive silencer element/repressor element-1(NRSE/RE-1).^89^-^92^ NRSE/RE-1 was originally discovered in the promoters of the genes that express SCG 10^93^ and the type II sodium channel,^94^ and later shown to mediate negative regulation of neuronal genes. Through binding to NRSE/RE-1, NRSF/REST represses multiple neuronal target genes in non-neuronal tissues and also in undifferentiated neural precursors of the CNS. Its function is to control the proper timing of neuronal gene expression during neurogenesis. To understand the role of negative regulation of neuronal gene expression by NRSF/REST, we investigated the effects of alcohol on the DNA binding activity of NRSF/REST. These experiments demonstrated that alcohol enhanced the NRSF/REST binding activity to NRSE/RE-1 in a concentration-dependent manner and these concentrations inhibited neuronal differentiation without affecting cell survival.^95^

We then investigated the molecular mechanisms that underlie the alcohol inhibition of neuronal differentiation, specifically the expression of ERK and its phosphorylated (active) form after alcohol exposure. The treatment of NSCs with alcohol decreased phosphorylation of ERK, whereas the expression of total-ERK was not affected. To confirm the involvement of ERK in the mechanism of alcohol inhibition of neuronal differentiation, we treated NSCs with U0126, a mitogen-activated ERK kinase (MEK) inhibitor. MEK locates upstream of ERK in the MAP kinase cascade and its suppression by U0126 decreases ERK expression. U0126 treatment of NSC reduced neuronal differentiation and decreased the generation of neurons.^95^ Furthermore, the effect of U0126 on the DNA binding activity of NRSF was measured by treating NSCs with various concentrations of U0126, which revealed that U0126 potentiated the NRSF binding activity at the same concentration which suppressed neurogenesis.

## Fetal Alcohol Effects and Neural Stem Cell Transplantation

A clinical entity called fetal alcohol effects (FAE) is a cluster of symptoms observed in children born to mothers with a history of heavy alcohol consumption during pregnancy.^96^-^98^ Fetal alcohol syndrome (FAS) is the severe form of FAE characterized by minor facial anomalies, prenatal and postnatal growth retardation, and cognitive and behavioral abnormalities.^99^,^100^ A large number of studies have demonstrated that compared to adult brain, the developing nervous system is more susceptible to ethanol toxicity.^81^,^101^-^104^ Although FAS/FAE is a completely preventable condition, once the children develop FAE, its effects are permanent and currently there is no fundamental treatment.

Studies on neurogenesis can be divided into two main clusters, namely, those focusing on the role of neurogenesis in brain function and those aiming to use these cells to treat degenerative disorders. The candidate medical conditions for cell transplantation therapy include brain injury,^105^ cerebral infarction,^106^ Parkinson's disease,^107^ Huntington's disease^108^ and multiple sclerosis.^109^ However, a PubMed search failed to find any reports of cell transplantation in models of alcohol-related disorders.

We transplanted NSCs into the FAE model rat to determine the potential for repair of the disrupted neural network and to explore the possibility of regenerative stem cell therapy for FAE.^110^ FAE model rats were prepared by administering a high dose of alcohol (4 g/kg/day, 4 days) to pregnant rats on gestational days 10 to 13.^111^ NSCs prepared from 13.5-day-old healthy rat embryos were stained with fluorescein-based dye to trace their migration and labeled with [35S]-methionine to quantify their migration into the brain. These NSCs were transplanted through the tail vein one month after the prenatal alcohol exposure. Behavioral analysis using an elevated plus maze was performed 40 days after the NSC transplantation and was followed by histological analysis of the brain. Transplanted NSCs were detected in wide areas of the brain, and the number of the cells in the brains of FAE rats was higher than in the control group. Furthermore, NSC transplantation reversed the behavioral abnormalities observed in the FAE rats, such as hyperactivity and decreased anxiety.^110^ We concluded that NSC transplantation is a potentially promising new strategy for the treatment of alcohol-related abnormalities in FAS/ FAE.

## Conclusion

Despite thousands of published studies on alcohol cytotoxicity, the true mechanism of alcohol-induced neuronal damage remains unclear. This review covers recent studies which prompt us to link neurogenesis to the pathophysiology of alcohol-related neuro-psychiatric conditions. There are a few studies which indicate an increase of hippocampal neurogenesis in certain conditions^74^,^112^-^114^ although most studies have demonstrated decreased neurogenesis. These conflicting results are perplexing although some do suggest the existence of factors which might be applied as novel treatment of alcohol-induced cell damage. Further studies of the mechanism of alcohol inhibition of neurogenesis are urgently needed and may open the door to new treatment strategies for alcohol-related disorders.
